# Supplementary material for: Systematic Comparison of FBS and Medium Variation Effect on Key Cellular Processes Using Morphological Profiling
Source: Cells. 2025 Feb 25;14(5):336. doi: 10.3390/cells14050336 (PMC11898771; doi:10.3390/cells14050336)
Supplement: Supplementary file 1 [file cells-14-00336-s001.zip › cells-3480757-supplementary/Supplementary.pdf]

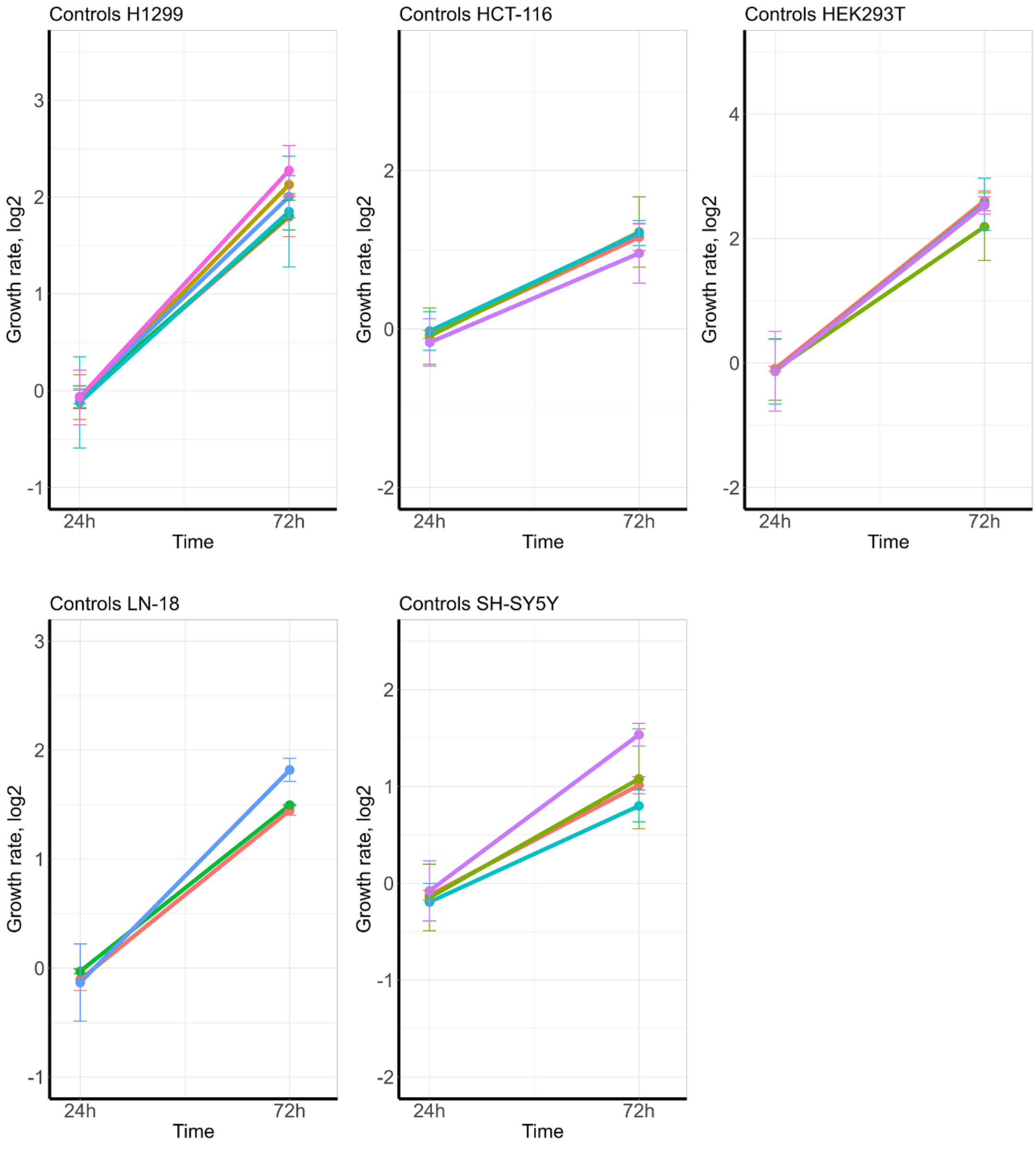

Figure S1. Cell proliferation for control conditions. Changes in growth rate are shown in log2 scale, growth rate was calculated as ratio of cell numbers at 72h or 24h relative to a mean for all control conditions.

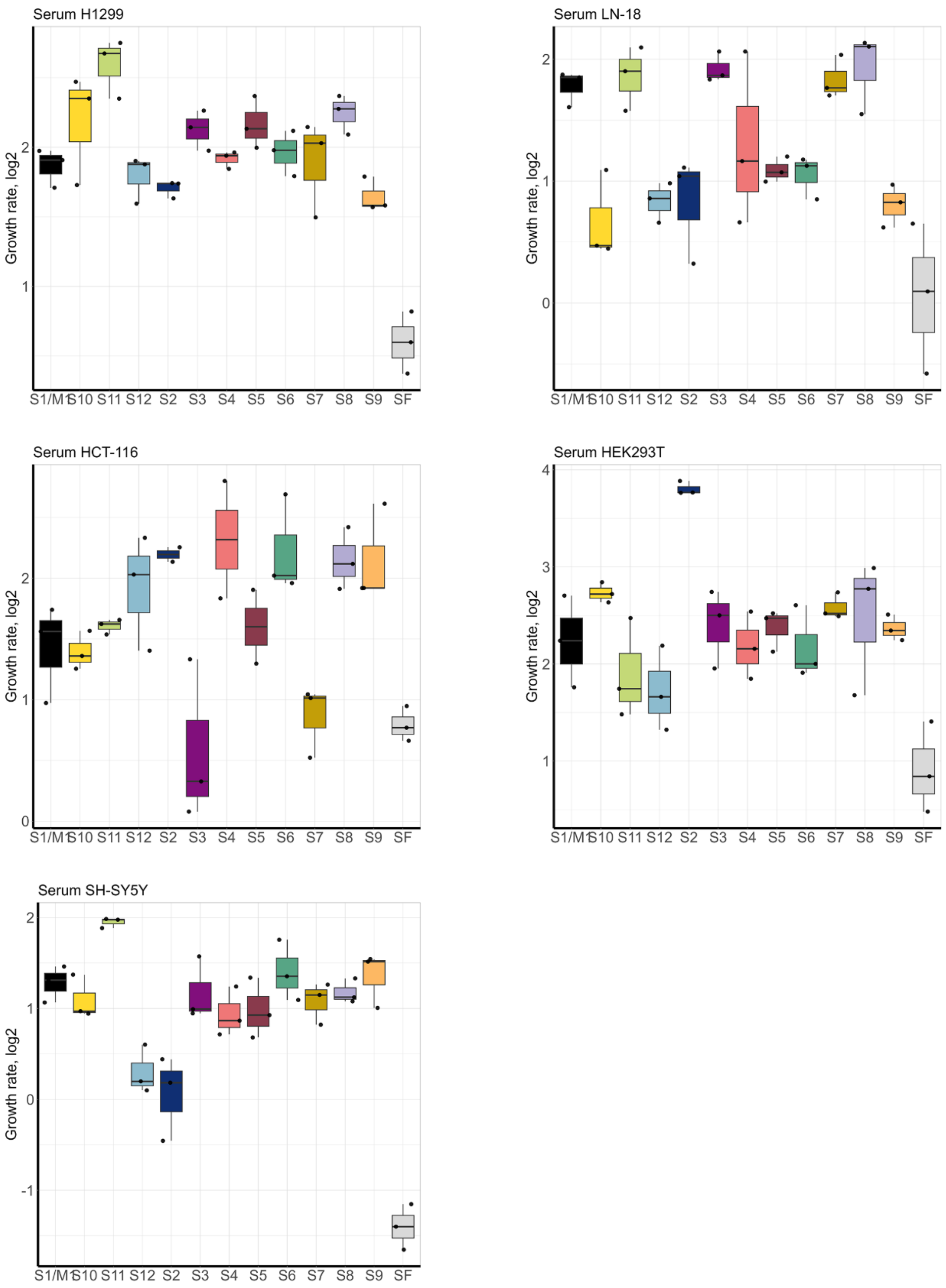

Figure S2. FBS variation effects on cell proliferation 72h after growth conditions change. Changes in growth rate are shown in log2 scale, growth rate was calculated as ratio of cell numbers at 72h or 24h relative to control condition (without growth media change). Points indicate data for repeats, box plot shows median, SD and min-max variation.

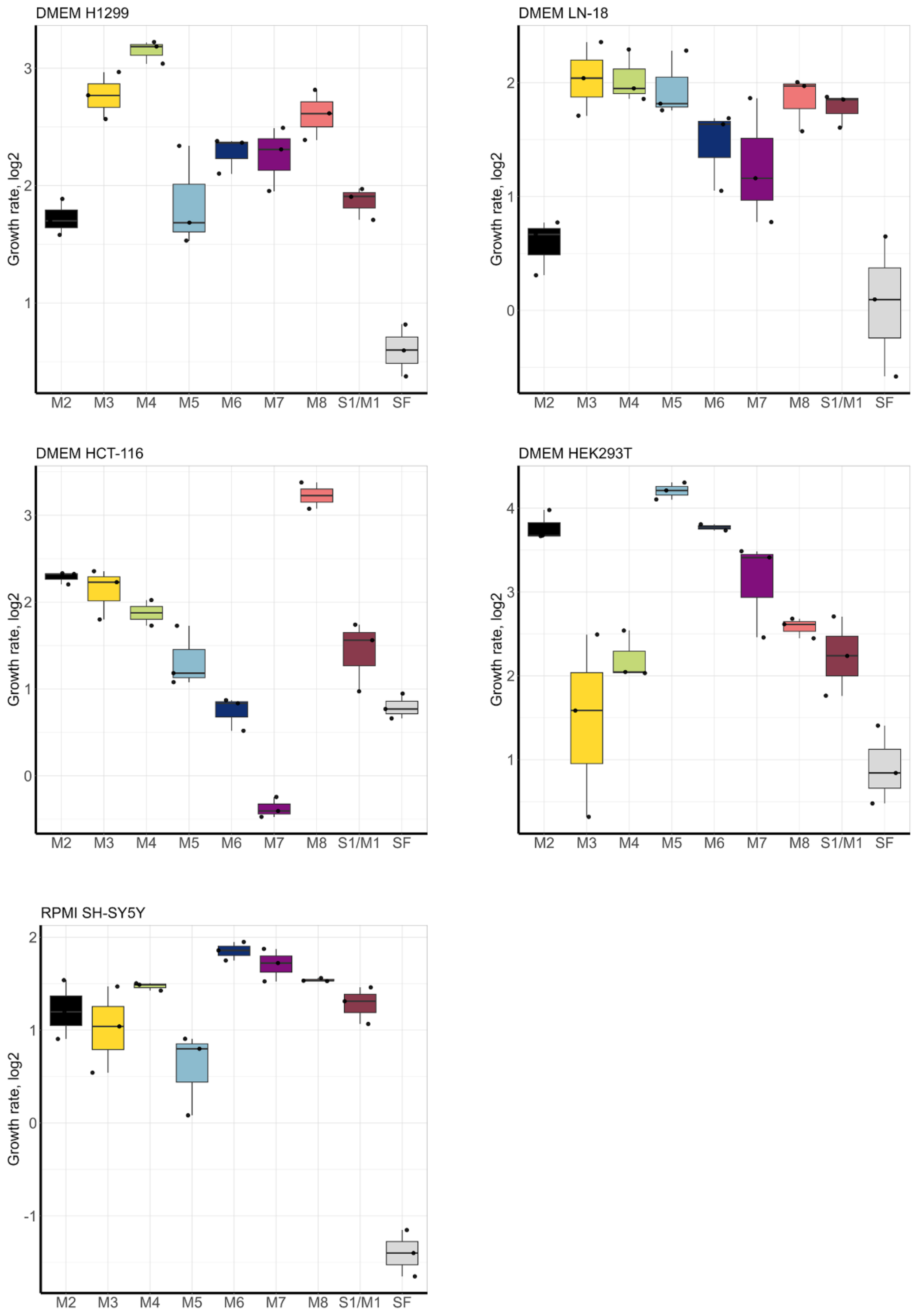

Figure S3. DMEM or RPMI variation effects on cell proliferation with 10% FBS 72h after growth conditions change. Changes in growth rate are shown in log2 scale, growth rate was calculated as ratio of cell numbers at 72h or 24h relative to control condition (without growth media change). Points indicate data for repeats, box plot shows median, SD and min-max variation.

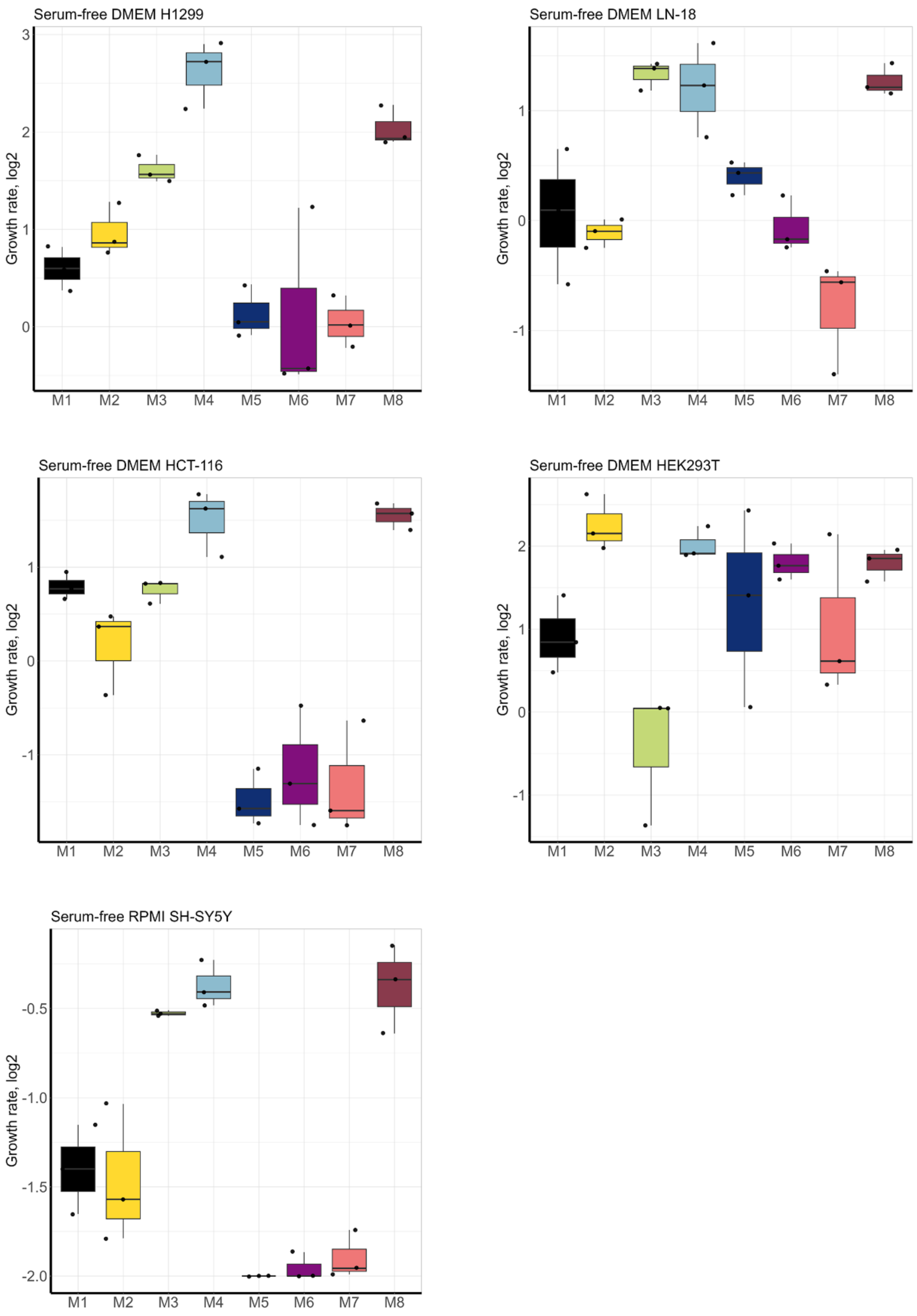

Figure S4. DMEM or RPMI variation effects on cell proliferation without FBS 72h after growth conditions change. Changes in growth rate are shown in log2 scale, growth rate was calculated as ratio of cell numbers at 72h or 24h relative to control condition (without growth media change). Points indicate data for repeats, box plot shows median, SD and min-max variation.

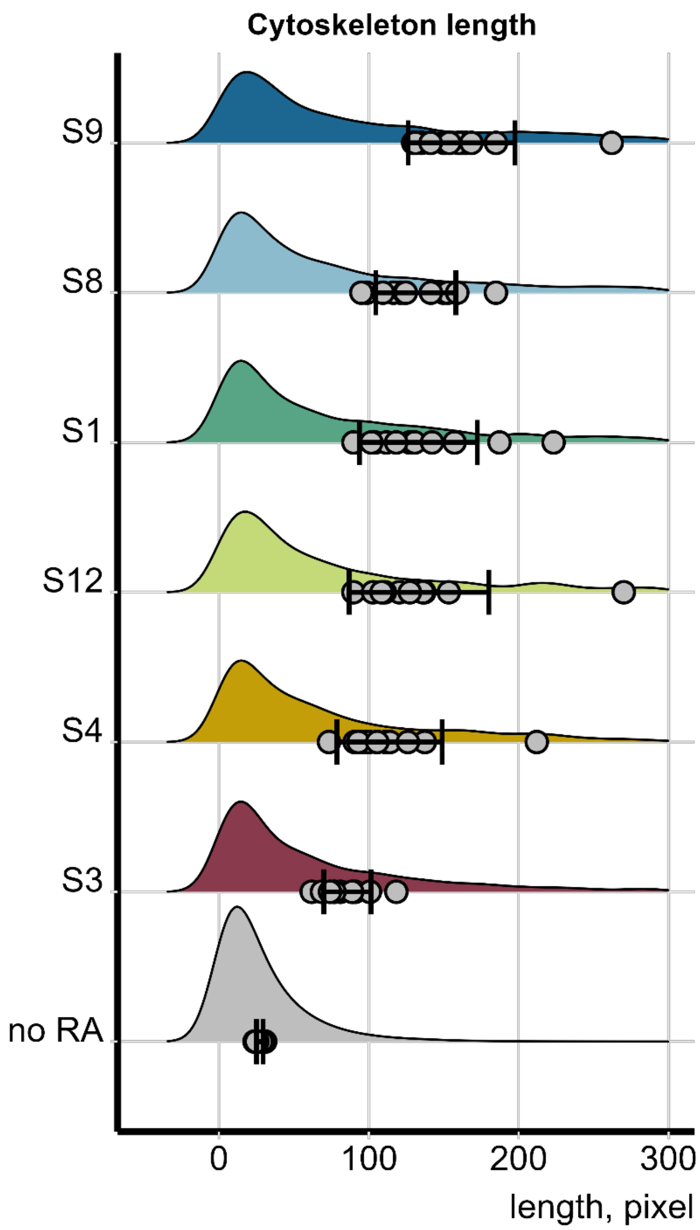

Figure S5. Distribution of cytoskeleton length of differentiated SH-SY5Y cells. Cells were differentiated with 10 uM retinoic acid in the presence of 5% FBS for 168h and then cytoskeleton length was measured in CellProfiler. Differentiated cells are compared with cells which were cultured with 5% FBS, but no retinoic acid (no RA).
